# A rapid realist review of clinical neuropsychology rehabilitation programmes to improve psychological wellbeing and quality of life for people with acquired brain injuries

**DOI:** 10.1080/09602011.2023.2273580

**Published:** 2023-11-17

**Authors:** K. Fletcher, S. Wydera, N. Thorpe, K. Radford, R. das Nair, V. Booth, on behalf of the NeRO Programme and Management Team

**Affiliations:** a Division of Rehabilitation & Ageing, University of Nottingham, Nottingham, UK; b Midlands Partnership NHS Foundation Trust, Stafford, UK; c Nottinghamshire Healthcare NHS Foundation Trust, Nottingham, UK; d SINTEF, Trondheim, Norway; e Institute of Mental Health, University of Nottingham, Nottingham, UK

**Keywords:** Neuropsychological rehabilitation, acquired brain injury, psychological wellbeing, quality of life, realist review

## Abstract

Approximately 20% of acquired brain injury (ABI) survivors experience reduced psychological wellbeing (PWB). Neuropsychological rehabilitation (NPR) is one approach supporting people with ABI to participate meaningfully in activities despite challenges. Although literature supports NPR effectiveness, little is known about change mechanisms. This systematic realist review identifies what NPR programmes have been designed, delivered, and evaluated for people with ABI to improve PWB and/or quality of life (QOL), as well as providing a context-relevant understanding of what NPR includes and how NPR might lead to positive outcomes. A rapid realist review was conducted in three phases: (1) structured retrieval and evidence extraction; (2) stakeholder consultation; (3) analysis and synthesis. Searches were completed, and findings from 35 publications and one stakeholder consultation were synthesized into a refined logic model. Six context-mechanism-outcome chains (CMOCs) were identified. Participants' relationships to internal experiences, and feelings of self-worth, mastery, and connection appeared to be mechanisms that led to improved PWB and QOL. Adaptation and individualized programmes were also key mechanisms to explain successful NPR. Embedding CMOCs into NPR could improve PWB and/or QOL for people with ABI. The logic model will inform ongoing development of a new online, group-based, NPR programme.

## Introduction

Acquired brain injury (ABI) caused by traumatic injuries (e.g., accidents) and non-traumatic conditions (e.g., stroke), result in over 1000 UK hospital admissions daily (Menon & Bryant, 2019). Some of the effects experienced by people following ABI include changes in cognition (e.g., issues with memory), reduced or changed physical abilities, fatigue, and behavioural changes (e.g., changes in personality and/or regulating emotions). Alone or combined, these can have a negative impact on participation in daily activities and chosen life roles, psychological wellbeing (PWB) and quality of life (QOL). Approximately 20% of ABI survivors experience reduced PWB (Hackett et al., 2005; Soo & Tate, 2007), alongside difficulties returning to work (Gadidi et al., 2011), challenges engaging in rehabilitation, increased disability (Whitnall et al., 2006), and suicide risk (Madsen et al., 2018). Consequently, societal cost of ABI-related disability can be significant because long-term or life-long support is often required (Turner-Stokes et al., 2015). Indeed, ABI costs the UK economy £15 billion annually (Parsonage, 2016), or 10% of the National Health Service (NHS) annual budget (HM Treasury, 2020), because of premature death, health and social care requirements, lost work contributions, and continuing disability.

One of the treatment options provided to ABI survivors to address resultant symptoms is neurological rehabilitation. Neurological rehabilitation can focus on various aspects of recovery, including physical, behavioural, cognitive, emotional, communication, and psychosocial changes (Wilson, 2008). Neuropsychological rehabilitation (NPR) aims to help people return to participation in meaningful activity despite challenges in multiple of the abovementioned aspects following ABI (Ben-Yishay, 1996; Wilson, 2008). NPR is a highly complex intervention based on brain-behaviour relationships and uses a rehabilitation paradigm to deliver interventions in direct, indirect, one-to-one or group settings. NPR programmes are often designed to respond to multiple factors, including brain injury location, systemic influences (e.g., family members), time post injury, functional impact, and person-centred goals. Although the evidence behind NPR is based primarily on face-to-face rehabilitation programmes (i.e., those conducted with the clinician and patient in the same physical room), online delivery options are now being utilized to increase patient choice and reduce exclusion due to extraneous factors (e.g., difficulty traveling to an outpatient setting). There is an implicit assumption that alternative modes/formats of intervention delivery (e.g., online formats) are as effective as in-person NPR and follow the same mechanisms of operation, but this has not been empirically studied. More research is, therefore, required to investigate helpful mechanisms in both face-to-face and alternatively delivered NPR interventions.

The combined cognitive and psychological outcomes linked to NPR have led to it being prescribed as an evidenced-based and routine treatment option for people with ABI in the NHS (SIGN, 2013). Although NPR is considered beneficial and effective at improving PWB (Aboulafia-Brakha & Ptak, 2016; Bertisch et al., 2011; Chouliara & Lincoln, 2016; Cicerone et al., 2011; Lundqvist et al., 2010; Mansson Lexell et al., 2013; Tulip et al., 2020; von Mesenkampff et al., 2015), the current evidence is yet to outline (i) the specific components that are indicated as leading to improvement (SIGN, 2013) or impact, and (ii) the most effective contextual features of NPR as an intervention. Furthermore, as there are no current online, group-based NPR available that have been systematically developed or evaluated, a systematic literature review that can explore these complex, unanswered questions to inform the development of a new online, group-based NPR is indicated.

Realist methodology is one approach that can take into consideration the complexity of NPR development and delivery. A realist review is a theory-led approach that applies principles of “realism” to systematic review (Pawson, 2006; Wong et al., 2013). Unlike traditional systematic reviews of interventions that typically aim to answer the question “Does it work?”, realist reviews aim to make sense of the relationship between a *context* (personal, organizational, social and/or policy factors) and mechanism (description of “*how the resources embedded in a program influence the reasoning and ultimately the behaviour of program subject*s’ p13; Pawson, 2013), and how this can lead to specific *outcomes* (intended and/or unintended result). The methodology assumes that interventions themselves do not lead to change rather, the interaction of resource, context, and human reasoning influences intervention outcome. Context-Mechanism-Outcome chains (CMOCs) illustrate the relationship between these interacting factors, which help to build and refine theory, which in the case of this review, will be presented as a logic model.

Although the current review could have focused on online group-based interventions in fields beyond NPR (e.g., mental health more broadly), our aim was to develop an explanatory account of the CMOCs that contribute to successful outcomes as a result of NPR for people with ABI, and translating these CMOCs to the online, group environment, and using the logic model developed to inform the next stage in the development of a new NPR programme.

It asks the following question:

What NPR programmes have been delivered and evaluated for people with ABI to improve PWB and/or QOL, what do they include, and how might they lead to success?

The sub-questions include: (1) In what context are NPR programmes delivered? (2) What are the intervention characteristics of NPR programmes? (3) What PWB and QOL outcome measures are used to evaluate NPR programmes? (4) What are the key CMOCs of NPR programmes?

## Materials and methods

To capture the complexity of neuropsychological rehabilitation, realist review methods were used to develop a theoretical understanding of the CMOCs underpinning successful NPR targeting PWB and QOL for people with ABI. The review followed three phases: (1) structured retrieval and extraction of the evidence; (2) stakeholder consultation; and (3) analysis and synthesis to build and refine a logic model. A complete account of the review protocol is available (Fletcher et al., 2022). The phases were not sequential, but overlapped, leading to building of evidence. The review search was structured into four levels, with the intention of accessing levels 2, 3 and 4 only if sufficient depth of information was not gathered at previous levels. The levels set out in the protocol included:

Level 1: (1) PWB and/or QOL is the primary outcome

Level 2: (1) PWB and/or QOL is the secondary outcome;

In addition, for both Level 1 and 2, NPR was delivered directly to the person with ABI by Clinical Psychologists, Neuropsychologists, and/or other mental health professionals working in NPR.

Level 3: (1) PWB and/or QOL of life is the primary outcome;

Level 4: (1) PWB and/or QOL is the secondary outcome;

In addition, for both Level 3 and 4, NPR was delivered to the person with ABI by the family or healthcare team.”

Although the review could have utilized multiple areas of evidence using different levels of inclusion criteria, a decision was made after level 1 full-text screening to limit the review to the Level 1 area of evidence. The rationale for this was that sufficient papers (*n* = 35) had been found to satisfy the intention of the review and limiting the included papers to Level 1 would allow the focus to remain on building a preliminary logic model to inform the next stage of intervention development in the restricted timeframe.

## Phases of the realist review

### Phase 1: Structured retrieval and extraction of the evidence

A systematic literature search was undertaken according to the Preferred Reporting Items for Systematic Reviews and Meta-Analyses (PRISMA) guide (Page et al., 2021). The following databases were searched in March 2022: Medline ALL, Embase, PsycINFO (all via Ovid), and Cochrane Library. The search was restricted to papers available in the English language and no publication date restrictions were applied. Supplementary website searching was conducted in Turning Research into Practice (TRIP), National Grey Literature Collection, and the JBI Systematic Review Register. Finally, JBI Evidence Synthesis journal was hand-searched from journal inception (2003) to March 2022.

The search strategy was undertaken by an information specialist (NT) using a combination of free text terms (searching the title, abstract, and author keywords where available) and relevant database specific controlled vocabulary headings, and advanced search syntax (truncation, Boolean logic AND/OR, and proximity searching) to ensure all relevant studies were identified. The search terms, which were co-developed with the wider study patient and public involvement group, included the following themes, with synonyms to describe each: stroke, neuropsychology rehabilitation, PWB and QOL.

Where PWB and QOL were included, the following theoretical background was considered: PWB is a multi-dimensional term encompassing feeling and functioning. Definitions include: (1) a state where individuals can realize their own potential to cope with life stressors and contribute to their community (WHO, 2004); (2) a state of feeling healthy and happy (Cambridge English Dictionary); (3) a multi-dimensional measurement encompassing autonomy, agency, life purpose, self-acceptance and interpersonal relationships (Ruggeri et al., 2020). Quality of Life is a multi-dimensional concept the reflects the wellbeing aspired to by an individual in domains including emotions, relationships, material wellbeing, personal development, physical function, self-determination, social inclusion and rights (Schalock et al., 2016). To capture psychological wellbeing and quality of life, primary outcome measures were thought to include those directly measuring wellbeing (e.g., the Warwick Edinburgh Mental Well Being Scale; Tennant et al., 2007), mood measures (e.g., Patient Health Questionnaire; Kroenke et al., 2001); anxiety measures (e.g., Hospital Anxiety and Depression Scale; Stern, 2014), acceptance measures (e.g., Acceptance and Action Questionnaire; Bond et al., 2011), self-compassion measures (e.g., Self-Compassion Scale; Neff, 2003), self-efficacy measures (e.g., Stroke Self-Efficacy Questionnaire; Riazi et al., 2014) and QOL measures (e.g., QOLIBRI; Von Steinbuchel et al., 2010)

The full details of the search strategy can be found in additional materials (Supplementary File 2).

### Study screening and data extraction

**Figure 1.**
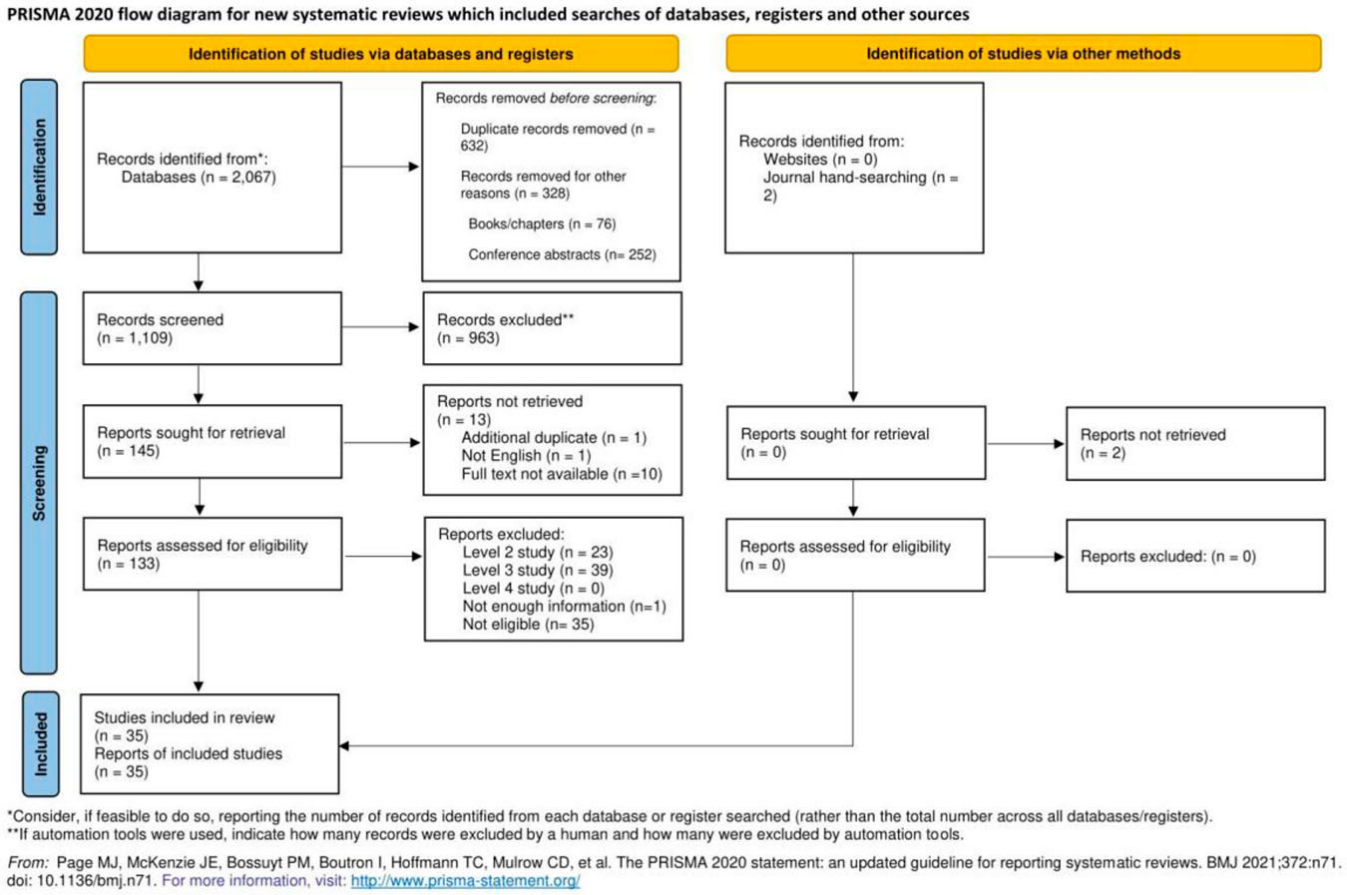
PRISMA flow chart.

References were imported into EndNote 20 reference software and duplicates removed. They were then imported into Rayyan online software for the screening of titles and abstracts. Two authors (KF and SW) screened all titles and abstracts. After 10% of abstracts were screened, reviewers consulted on any differences in opinion. After reaching agreement on inclusion/exclusion of all the papers based on titles and abstracts, full texts were retrieved. Out of 1,109 screened papers, 145 full texts were retrieved (see Figure 1). The same two reviewers screened all the full texts, and categorized articles (Levels 1–4) according to the study protocol.

As per Level 1 criteria, NPR programmes were included where: (1) PWB and/or QOL is the primary outcome; and (2) they were delivered directly to the patient by Clinical Psychologists, Neuropsychologists, and/or other mental health professionals working in NPR. NPR programmes were excluded if they were delivered to or by the family or neurological rehabilitation team (outside of those listed above). Where there was ambiguity about who delivered the NPR, studies were included if they were relevant to the review question.

Data were extracted by two reviewers (KF and SW) using a bespoke data extraction form designed to capture study, sample, and intervention characteristics. TIDieR characteristics (Cotterill et al., 2018) and CMOCs were extracted from the included studies by the reviewers. TIDieR checklist is a framework that provides structure and allows for keeping record of intervention development and details, including information on target population, delivery, infrastructure and required materials, as well as modifications and tailoring to the intervention (Cotterill et al., 2018). Since its creation in 2014, the TIDieR checklist has been widely used alongside different stages of intervention research and allows for relatively accurate evaluation of interventions in context of delivery (Cotterill et al., 2018). Furthermore, TIDieR checklist has been approved and used in systematic reviews as part of data extraction process and to assess quality of reporting interventions (Hoffmann et al., 2017; Jones et al., 2016; McMahon et al., 2015). Considering the variable quality of intervention detail reported in the papers and previous systematic reviews, the TIDieR checklist was deemed the most appropriate tool for intervention description for this review. The data extraction form based on the TIDieR checklist was piloted by KF and SW and shared with the team for comment. The CMOCs were not necessarily identified as such in the original publication and interpretation was required by the reviewers. To support the extraction of CMOCs and reduce potential for bias during extraction, 10% of forms were shared with co-author VB to appraise the extraction process and identified data.

Traditional critical appraisal tools for quality assessment typically used in systematic reviews were not used due to the pursuit of adhering to realist review principles. Following realist review methodology and the reporting standards and concepts for realist evaluation (Dada et al., 2023; Wong et al., 2016), the assessment of relevance and rigour of the studies was used to determine quality of the included texts. Relevance, defined as “whether [the data] can contribute to theory building and/or testing” (Wong et al., 2014), and rigour, indicating whether data are “trustworthy” or credible (Wong et al., 2014), thus describing the robustness of the methodology used and credibility of conclusions and inferences described by the authors, were quantified using a five-point scale (where: 1 = none whatsoever, 2 = poor, 3 = fair, 4 = good, 5 = exceptional) (based Brown et al., 2021). The scores attributed to each study were subjectively given based on the scoring above, for example studies that involve well designed empirical research on participants would be given a rigour rating of 5, and studies that were deemed to be extremely relevant to the goals of the realist review were given a relevance rating of 5. To reduce potential for bias, a known risk of accelerated reviews (Grant & Booth, 2009), 10% of relevance and rigour reviews were discussed between KF, SW, and VB to align approach and differences. Relevance and rigour ratings were used to guide the authors during development of the logic model, however consistent with literature encouraging use of multiple sources in realist reviews (Pawson, 2013), relevance or rigour ratings did not impact inclusion in the review.

### Phase 2: Stakeholder consultation

To maximize the relevance of the review for the reader and professionals working in NPR, two stakeholders were consulted for their expertise (Wong et al., 2014). Stakeholders included a Clinical Psychologist working with neurological populations and a Neuropsychologist, each with expertise in psychological models (e.g., cognitive-behaviour therapy, acceptance and commitment therapy). Stakeholder consultation was facilitated by two review authors (KF and RdN) on one occasion, and identified mechanisms were discussed. This discussion was recorded and transcribed verbatim., with ethical approval obtained from the University of Nottingham Division of Psychiatry Ethics Committee (reference number 2897). Data were analyzed using framework analysis (Gale et al., 2013), informed by the consolidated framework for implementation research (Damschroder et al., 2009). Evidence was used to: (1) determine possible theoretical assumptions about how and why NPR programmes targeting PWB and/or QOL work or not; (2) develop or refute provisional proposed mechanisms determined by the literature as leading to successful NPR programmes. Although further iterative rounds of discussion are planned within the intervention development, the stakeholders outlined here were not further involved in the development of emerging CMOCs or building the logic model at this stage of the intervention development.

### Phase 3: Analysis and synthesis

Data synthesis was led by KF and emerging findings, including interpreted CMOCs and challenges to interpretation, were shared and discussed with the team (SW and VB) through a series of meetings. Discussion assisted the refining of CMOC interpretations, ensuring clarity, and a clear audit trail to interpretation. Discussion of papers included characteristics of the delivered NPR, resources, and outcomes. The focus of the data synthesis was to understand what mechanisms are responsible for improving PWB and/or QOL outcomes in people with ABI and in what contexts these mechanisms are present or activated. Recurring mechanisms were grouped through thematic tabling, leading to the grouping of CMOCs according to the activated mechanism. The team then developed broad “if–then” statements to capture the CMOCs (Astbury, 2018). The final synthesis was displayed in tabular, narrative and figure format, resulting in a preliminary logic model.

## Results

### Phase 1: Review

After removal of duplicates and article screening, 145 papers were considered for inclusion. In total, 35 studies investigating NPR programmes targeting PWB and/or QOL as primary outcomes for people after ABI were included in the final synthesis.

### In what context are NPR programmes targeting PWB and/or QOL for people after ABI delivered?

Key contextual factors were extracted for all studies (see Table 1). Of the included papers, studies completed in the USA (study number 2, 4-6, 16, 20, 21, 24, 35 in Table 1, Table 2 and Table 3), UK (1, 3, 15, 19, 26, 30, 31), and Netherlands (13, 18, 27-29, 33) were most frequent (26%, 20% and 17% respectively), with fewer studies being reported from Canada (7, 9, 23), New Zealand (8), Spain (10), Italy (11) Germany (12), Australia (14, 17) Indonesia (22), Nigeria (25), Switzerland (32) and China (34). There were 83% of studies completed in an outpatient setting (1-13, 15, 16, 18-23, 25-30, 32-34), with the remainder being home-based (14, 31, 35), hospital and community (17), and inpatient (24). The design of the studies was variable, with 48.6% being randomized controlled trials (2, 6, 9, 12, 13, 17-21, 25, 26, 31-34), 20% being single case, or single case series designs (1, 3, 14, 24, 27, 29, 30), 14% pre–post studies (4, 15, 22, 23, 35), and the remaining an active controlled trial (5), matched controlled trial (7), non-randomized wait list controlled trial (8), parallel group randomized trial (10, 16), cross over trial (11), and a prospective cohort study (28).

**Table 1.** Context & characteristics of NPR.

Study No	Authors	Country	Healthcare setting	Study design	Sample**CSBARLINE** Age: Mean (SD)**CSBARLINE** Male: X%	ABI Diagnoses Investigated (CSITALICSTARTAVCSITALICEND time since injury)
1	Addy (2011)	UK	OP	Case study	*n* = 1 Age: 51 Gender = F	SAH
2	Ashman et al. (2014)	USA	OP	RCT	*n* = 77 Age: 47.1 (10.6) – 48.1 (20.2) Male: 41%	TBI
3	Ashworth et al. (2011)	UK	OP	Case study	*n* = 1 Age: 23 Male: 0%	TBI
4	Azulay et al. (2013)	USA	OP	Pre-post study	*n* = 22 Age: 48.9 (8.3) Male: 50%	TBI
5	Bay and Chan (2019)	USA	OP	Active-controlled trial	*n* = 33 Age: – Male: 32%	TBI
6	Bomyea et al. (2017)	USA	OP	RCT	*n* = 129 (n_ABI _= 83) Age: 34.05 (7.65) – 35.27 (8.81) Male: 78%	TBI
7	Bradbury et al. (2008)	Canada	OP	Matched-controlled trial	*n* = 20 Age: 39.8 (10.44) – 42.5 (13.01) Male: 50%	Aneurysm Hypoxia TBI Stroke
8	Chalmers et al. (2019)	New Zealand	OP	Non-randomised waitlist-controlled trial	*n* = 29 Age: 56.34 (9.57) Male: 58.6%	Stroke
9	Cisneros et al. (2021)	Canada	OP	Semi-RCT	*n* = 37 Age: 63.75 (5.63) – 64.9 (7.18) Male: 59%	TBI
10	Cuberos-Urbano et al. (2018)	Spain	OP	Randomised parallel-group pilot trial	*n* = 16 Age: 32.24 (10.75) Male: 88%	TBI
11	Di Vita et al. (2022)	Italy	OP	Cross-over trial	*n* = 9 Age: 34.11 (12.44) Male: –	TBI
12	Exner et al. (2021)	Germany	OP	Randomised waitlist-controlled trial	*n* = 56 Age: 44.2 (11.9) – 47.0 (10.2) Male: 54%	TBI Vascular brain damage Other
13	Gehring et al. (2009)	Netherlands	OP	Randomised waitlist-controlled trial	*n* = 140 Age: 42.0 (9.4) – 43.8 (10.5) Male: 57.9%	Brain tumour
14	Gertler & Tate (2021)	Australia	Home	SSED	N = 3 Age: 32.7 (11.5) Male: 100%	Stroke TBI
15	Glazer et al. (2022)	UK	OP	Pre-post feasibility study	*n* = 19 Age: – Male: –	Brain tumour
16	Hart, Vacarro, Collier, Chervoneva, & Fann (2020)	USA	OP	Parallel-group randomised trial	N = 59 Age: 38.5 (15.3) – 40.4 (15.2) Male: 79.7%	TBI
17	Hoffmann et al. (2017)	Australia	Hospital + community	Pilot RCT	*n* = 33 Age: 57.0 (14.2) – 63.6 (13.0) Male: 66.7%	Stroke
18	Kootker et al. (2017)	Netherlands	OP	RCT	*n* = 61 Age: 61 [median] Male: 62.3%	Stroke
19	Majumdar and Morris (2019)	UK	OP	RCT	*n* = 53 Age: 62.7 (13.9) Male: 60.4%	Stroke
20	Milbury et al. (2020)	USA	OP	RCT	*n* = 22 [couples] Age: 52.12 (10.95) – 57.67 (12.18) Male: 48.6%	Brain tumour
21	Mitchell et al. (2009)	USA	OP	RCT	*n* = 101 Age: 57 Male: 60.4%	Stroke
22	Mohamad et al. (2020)	Indonesia	OP	Pre-post study	*n* = 60 Age: – Male: 48.3%	Stroke
23	Moustgaard (2005)	Canada	OP	Pre-post study	*n* = 32 (n_ABI _= 23) Age: – Male: –	Stroke
24	Olive (2001)	USA	IP	Case study series	*n* = 5 Age: 60.6 (12.1) Male: 40%	Stroke
25	Olukolade & Osinowo (2017)	Nigeria	OP	RCT	*n* = 30 Age: – Male: 43.3%	Stroke
26	Potter et al. (2016)	UK	OP	Randomised waitlist-controlled trial	*n* = 46 Age: 41.4 (11.6) Male: 54%	TBI
27	Rasquin et al. (2009)	Netherlands	OP	SSED	*n* = 5 Age: 46.2 (5.5) Male: 20%	Stroke
28	Rasquin et al. (2009)	Netherlands	OP	Prospective cohort study	*n* = 27 Age: 49.5 (9.2) Male: 52%	Stroke TBI
29	Rauwenhoff et al. (2022)	Netherlands	OP	Case study series (non-concurrent multiple baseline design)	*n* = 4 Age: 51.0 (12.6) Male: 75%	TBI Stroke
30	Roche (2020)	UK	OP	Case study	*n* = 1 Age: 48 Male = 0%	TBI
31	Thomas et al. (2019)	UK	Home	Parallel-group RCT	*n* = 39 Age: 65.8 (11.3) Male: 61.5%	Stroke
32	Urech et al. (2020)	Switzerland	OP	RCT	*n* = 25 Age: 45.6 (12.6) – 50.8 (7.8) Male: 44%	Brain tumour Encephalitis Stroke TBI
33	van Eeden et al. (2015)	Netherlands	OP	RCT	*n* = 61 Age: 60.0 (10.5) – 62.2 (8.3) Male: 62.3%	Stroke
34	Wang et al. (2020)	China	OP	Randomised active-controlled trial	*n* = 134 Age: 59.9 (10.7) Male: 46.3%	Stroke
35	Wathugala et al. (2019)	USA	Home	Pre-post study	*n* = 10 Age: 59.8 Male: 90%	Stroke

OP = outpatient; IP = inpatient; NS = Not specified, RCT = randomised controlled trial, SAH = subarachnoid haemorrhage; TBI = traumatic brain injury; SSED = Single-subject experimental design.

**Table 2.** Outcome measures used to investigate PWB and/or QOL.

Study No	Authors	Psychological wellbeing outcomes targeting mood or anxiety evaluated	Quality of life outcomes evaluated
1	Addy (2011)	-	Qualitative subjective self-reported quality of life*
2	Ashman et al. (2014)	Structured Clinical Interview for DSM-IV depressive mood disorder*; Anxiety Disorder and Substance Abuse Modules of the Structured Clinical Interview for DSM-IV*; Beck Depression Inventory-II (BDI-II) State-Trait Anxiety Inventory (STAI) Interpersonal Support Evaluation List (ISEL) Life Experiences Survey (LES)	Life-3
3	Ashworth et al. (2011)	Beck Anxiety Inventory (BAI) BDI-II	–
4	Azulay et al. (2013)	Physical Self-Efficacy Scale (PSES) Mindful Attention Awareness Scale (MAAS)	Perceived Quality of Life (PQOL)
5	Bay and Chan (2019)	Center for Epidemiologic Studies Depression Scale (CES-D)	–
6	Bomyea et al. (2017)	Brief Symptom Inventory (BSI-18) Short Form 12 Survey – Mental Composite Score (SFMCS-12)	–
7	Bradbury et al. (2008)	Symptom Checklist-90-Revised (SCL-90-R) Depression, Anxiety and Stress Scale (DASS-21) Ways of Coping Questionnaire – Revised (WCQ-R)	–
8	Chalmers et al. (2019)	CES-D Hospital Anxiety and Depression Scale – Anxiety subscale (HADS-A)	Stroke-Specific Quality of Life Scale (SSQOL)
9	Cisneros et al. (2021)	Psychological General Well-Being Index (PGWBI)	–
10	Cuberos-Urbano et al. (2018)	–	Quality of Life after Brain Injury (QOLIBRI)
11	Di Vita et al. (2022)	BDI-II STAI	QOLIBRI
12	Exner et al. (2021)	Symptom Checklist-90-Revised (SCL-90-R), Global Severity Index (GSI) CES-D Positive and Negative Affect Schedule (PANAS) Community Integration Questionnaire (CIQ)	Schedule for the Evaluation of Individual Quality of Life-Direct Weighting (SEIQOL-DW) QOLIBRI
13	Gehring et al. (2009)	–	Short Form-36 (SF-36)
14	Gertler & Tate (2021)	Single-item Mood Likert Scale DASS-21 Rosenberg Self-Esteem Scale (RSES)	QOLIBRI Satisfaction With Life Scale (SWLS)
15	Glazer et al. (2022)	Qualitative feedback*	–
16	Hart et al. (2020)	Behavioural Activation for Depression Scale (BADS) BSI GSI Patient Health Questionnaire (PHQ-9) Generalised Anxiety Disorder Scale (GAD-7)	–
17	Hoffmann et al. (2017)	Montgomery – Åsberg Depression Rating Scale (MADRS) HADS Stroke Self-Efficacy Questionnaire (SSEQ)	Stroke and Aphasia Quality of Life Scale (SAQOL)
18	Kootker et al. (2017)	HADS Post-Stroke Depression Scale (PSDRS) Utrecht Proactive Coping Competence (UPCC)	SSQOL
19	Majumdar and Morris (2019)	PHQ-9 GAD-7 The Warwick-Edinburgh Mental Wellbeing Scale (WEMWBS)	EQ-5L-5D
20	Milbury et al. (2020)	CES-D	–
21	Mitchell et al. (2009)	Hamilton Depression Rating Scale (HDRS)	–
22	Mohamad et al. (2020)	–	World Health Organization Quality of Life Brief Version (WHOQOL-BREF)
23	Moustgaard (2005)	BAI BDI-II MAAS	SSQOL SF-36 Instrumental Activities of Daily Living (IADL) Dysfunctional Behaviour Rating Instrument (DBRI) Health-Promoting Lifestyle Profile (HPLP)
24	Olive (2001)	Nurse and caregiver mood ratings*	-
25	Olukolade & Osinowo (2017)	BDI Life Event Stress Scale (LESS)	–
26	Potter et al. (2016)	HADS Impact of Events Scale – Revised (IES-R) State-Trait Anger Expression Inventory-2 (STAXI-2) Brain Injury Community Rehabilitation Outcome Scale (BICRO-39)	Quality of Life Assessment Schedule (QOLAS) European Quality of Life (Euro-QOL) from EQ-5D
27	Rasquin et al. (2009)	BDI-II	–
28	Rasquin et al. (2009)	–	Stroke-Adapted Sickness Impact Profile (SA-SIP)
29	Rauwenhoff et al. (2022)	HADS	SF-12
30	Roche (2020)	HADS	EQ-5D
31	Thomas et al. (2019)	PHQ-9 Visual Analogue Mood Scales (VAMS) Sad	EURO-QOL-5
32	Urech et al. (2020)	BDI-II	WHOQOL-BREF
33	van Eeden et al. (2015)	HADS	EURO-QOL
34	Wang et al. (2020)	CES-D	Functional Assessment of Cancer Therapy – Brain (FACT-Br)
35	Wathugala et al. (2019)	HADS	SSQOL

* = qualitative measures.

**Table 3.** CMOCs identified.

CMOC	Contexts	Mechanisms	Outcomes (for NPR Group)	References
1	Interventions that focus on thoughts	Relationship with internal experiences	Reduced depression Reduced self-criticism & avoidance Deeper understanding of psychological factors related to rehabilitation Reduced neurobehavioral symptoms	Ashworth et al. (2011) Azulay et al. (2013) Bomyea et al. (2017) Di Vita et al. (2022) Majumdar and Morris (2019) Mitchell et al. (2009) Olive (2001) Rauwenhoff et al. (2022) Roche (2020) Urech et al. (2020) Wang et al. (2020)
2	Interventions that focus on developing a compassionate view of oneself	Self-worth	Enhanced self-validation and acceptance Positive changes in emotional well-being and self-esteem Reduced stress	Ashworth et al. (2011) Bay and Chan (2019)
3	Interventions that teach strategies to manage cognitive and emotional challenges	Mastery	Improved anxiety and depression Enhanced symptom management and symptom presentation Increased self-efficacy, self-control & self-regulation Mixed impact on QOL	Addy (2011) Bay and Chan (2019) Cisneros et al. (2021) Cuberos-Urbano et al. (2018) Gehring et al. (2009) Glazer et al. (2022) Rauwenhoff et al. (2022) Urech et al. (2020)
4	Interventions that include others	Connection	Reduced depression Spontaneous peer support, reduced isolation and loneliness Reduced avoidance Reduced physical symptoms	Cisneros et al. (2021) Cuberos-Urbano et al. (2018) Glazer et al. (2022) Milbury et al. (2020) Rauwenhoff et al. (2022)
5	Interventions that consider cognitive and psychosocial characteristics of the target population	Adaptation	Within group improvement on depression measures Improved QOL	Ashman et al. (2014) Bomyea et al. (2017) Cuberos-Urbano et al. (2018) Rasquin et al. (2009) Urech et al. (2020) Wathugala et al. (2019)
6	Interventions that incorporate a responsive facilitator	Individualised	Improved intervention impact for individual participants Higher? participant satisfaction Improved quality of life Reduced generalisability	Cuberos-Urbano et al. (2018) Exner et al. (2021) Potter et al. (2016) Roche (2020) Urech et al. (2020) van Eeden et al. (2015)

Studies investigating participants with stroke and traumatic brain injury (TBI) dominated the literature (40% and 34% respectively; 2-12, 14, 16-19, 21-35). Brain tumour populations were investigated in 11% of studies (13, 15, 20, 32) and hypoxia (7) and encephalitis (32) populations were investigated on only one occasion and as part of a wider sample (3%). There were three studies investigating vascular brain injuries (1, 7, 12). Other conditions considered under the umbrella term of ABI, for example meningitis and toxic or metabolic injury, were not represented.

### What are the intervention characteristics of NPR programmes targeting PWB and/or QOL for people after ABI?

Intervention characteristics, according to the TIDieR (Cotterill et al., 2018) checklist are presented in Supplementary File 3. The “how well” part of the checklist has been populated using limitations identified by the authors of each paper, as well as relevance and rigour ratings. Interventions delivered utilized a range of materials and adaptations. Materials included therapy manuals, workbooks or education (4, 6, 10, 12, 14-17, 19, 20, 31, 32 in Table 1, Table 2 & Table 3), pre-recorded audios and/or apps (e.g., for mindfulness; 27, 29, 30, 35), mood tracking (24) and recordings for participants who could not attend (5). Adaptations included memory supports (2) and recaps (12), lifelog recordings (10), diagrams and visual aids (3, 15), written handouts (2, 5), personalized action plans (33) and homework manuals or programmes (4, 13, 23, 26) with prompts or reminders to complete (12, 16). Tailoring and modifications were reported as occurring in 27/35 studies (77%; 1-10, 12, 15-19, 21, 23, 26-33, 35).

Facilitators of interventions were primarily psychologists (including assistant and trainee psychologists) and neuropsychologists (57%; 1, 3, 4, 7, 8, 12-15, 18, 19, 26-33), but also included experienced post-doctoral fellows and research team members (2, 10, 35), mental health professionals (5), therapists and/or counsellors (6, 10, 16, 20, 23, 24, 34), nurses (21), and health professionals recruited to deliver psychological intervention (17).

When considering “how” or intervention method, face-to-face (in-person sessions) was by far the most common intervention delivery method (80%; 1-4, 6-12, 14, 15, 17-19, 21, 23-34), with only one study completed solely in an online format (3%; 20), and four studies reporting a mixed delivery format (11%; 5, 13, 16, 35). Groups were used for intervention in 34% of studies (4, 5, 7, 8, 10, 11, 15, 19, 23, 25, 28, 34), with the majority conducted on an individual basis (63%; 1-3, 6, 7, 9, 12, 13, 14, 16-18, 21, 24, 26, 27, 29-33, 35). One study reported providing intervention to couples (3%; 20). The range of NPR sessions varied from 1 to up to 80 sessions, delivered from as often as daily to monthly. However, a couple of studies did not report this level of detail (11, 22). Session length varied; the shortest sessions lasted 30 min, and the longest lasted 150 min.

There were 10 studies that were exceptionally relevant to this review (29%; 2-7, 12, 21, 26, 32, 34), 17 of good relevance (49%; 6, 8, 9, 13, 15, 16, 17, 19, 20, 23, 25, 27, 28-31, 35), 4 of fair relevance (11%; 10, 11, 14, 24), and 4 of poor relevance (11%; 1, 18, 22, 33). Four studies were determined to present an exceptional level of rigour (11%; 19, 21, 26, 34). 16 studies presented good level of rigour (46%; 2, 5-9, 12, 13, 15-18, 25, 31-33). Fair level of rigour was observed in 8 studies (23%; 1, 4, 10, 14, 20, 23, 24, 29), and 6 studies presented poor rigour (17%; 11, 22, 27, 28, 30, 35D). Very poor rigour was determined in one study (3%; 3).

### What PWB and QOL outcome measures are used in the evaluation of NPR for people after ABI?

Within the 35 studies included, 54 quantitative outcome measures were used to capture PWB and/or QOL. A wider variety of PWB measures was used (*n* = 37, 69%; 2-9, 11, 12, 14-16, 18-21, 23-27, 29-35) when compared to the number QOL measures (*n* = 17, 31%; 1, 2, 4, 8, 10-14, 17-19, 22, 23, 26, 28-33) (See Table 2). There were 35 PWB measures of previously published/tested scales. The remaining 2 comprised single-item mood Likert scale (14) and mood ratings provided by nurses and caregivers (24). Majority of QOL measures (16/17) were previously published/tested QOL scales.

Qualitative outcomes were also used in three of the studies, and included structured clinical interviews for depressive mood disorder, anxiety disorder and substance abuse (2), as well as qualitative feedback regarding changes in psychological wellbeing (15) and self-reported subjective quality of life (1).

### What are the key CMOCs of NPR programmes targeting PWB and/or QOL for people after ABI?

Evidence from the 35 included papers led to the development of six CMOCs related to the delivery of NPR to people after ABI (see Table 3). The CMOCs are interrelated and the supporting evidence for each is discussed narratively below. All quotes included are taken directly from research papers.

CMOC 1: If interventions focusing on thoughts are delivered, then participants will relate to their internal experiences differently, leading to improvements in wellbeing

Changing the way people with ABI self-related to thought processes and/or emotional suffering was frequently reported as a mechanism related to improvements in wellbeing (Ashworth et al., 2011; Azulay et al., 2013; Bomyea et al., 2017; Di Vita et al., 2022; Majumdar & Morris, 2019; Mitchell et al., 2009; Olive, 2001; Rauwenhoff et al., 2022; Roche, 2020; Urech et al., 2020; Wang et al., 2020). The literature suggests that by addressing the way in which participants respond to themselves and/or their internal experiences, they learn to respond differently to the threat system, regulating self-critical thoughts and enhancing ability to tolerate distress.

Interventions where participants were supported to restructure cognitions and respond adaptively to thoughts included Neuropsychological Psychotherapy (Urech et al., 2020), Cognitive Behaviour Therapy (CBT; Olive, 2001), Acceptance & Commitment Therapy (ACT, Bomyea et al., 2017; Majumdar & Morris, 2019; Rauwenhoff et al., 2022; Roche, 2020), and Compassion Focused Therapy (CFT, Ashworth et al., 2011). Other approaches thought to produce similar mechanisms included psychosocial-behavioural treatment (e.g., pleasant event scheduling, problem-solving) combined with anti-depressant treatment (Mitchell et al., 2009), mindfulness-based interventions (e.g., mindful meditation, Bay & Chan, 2019; Wang, Li, Wang, & Lv, 2020), and art therapy (Di Vita et al., 2022).

Outcomes of the studies where this mechanism was activated were consistently positive across RCTs (Bomyea et al., 2017; Majumdar & Morris, 2019; Mitchell et al., 2009; Urech et al., 2020 Wang et al., 2020) and less rigorous research designs (Ashworth et al., 2011; Olive, 2001; Rauwenhoff et al., 2022; Roche, 2020). Outcomes indicted include reduced self-criticism (Ashworth et al., 2011), focus on previously avoided activity (Ashworth et al., 2011; Rauwenhoff et al., 2022) and movement towards a meaningful life (Majumdar & Morris, 2018), and a deeper understanding of the interaction of psychological factors in rehabilitation (Olive, 2001). Two RCTs reported significantly lower depression scores compared to a control group (Majumdar & Morris, 2019; Mitchell et al., 2009), and Urech et al. (2020) demonstrated a within-group decrease in depression scores for neuropsychological treatment and neuropsychological treatment plus psychotherapy, both immediately and at follow-up. One study, which targeted self-efficacy, reported a modest reduction in neurobehavioral symptoms (Azulay et al., 2013).

CMOC 2: If interventions develop a compassionate view of self, self-worth is activated, leading to improved PWB, and self-esteem

Two detailed interventions papers describing a positive-focused mindfulness group (Bay & Chan, 2019) and CFT (Ashworth et al., 2011), indicated positive outcomes when activating a self-worth mechanism. In an RCT, Bay and Chan (2019) reported fostering a compassionate view of oneself as part of the intervention. Similarly, in a single case study, Ashworth et al. (2011) discussed interventions focused on regulating the soothing system. Strategies used across both interventions include self-soothe training (e.g., “the perfect nurturer”), behaviour experiments, compassionate reframing, and mindfulness techniques, such as mindful movement, positive body awareness, and mindful and compassionate meditation. Ashworth et al. (2011) reported that when self-worth is activated, self-validation and acceptance is enhanced (participant feeling “much lighter within herself”; Ashworth et al., 2011, p. 136). Bay and Chan (2019) supported this single case study, reporting significant mean reductions in in stress and emotional challenges for participants.

CMOC 3: If participants are taught strategies to manage cognitive and emotional challenges, they are more likely to master them, and this will lead to positive wellbeing and improved symptom management

The literature indicates that building time into interventions to teach and practice strategies (e.g., within sessions or via structured work between sessions), led to a sense of mastery for people after ABI, whether these were group-based (Azulay et al., 2013; Bay & Chan, 2019; Chalmers et al., 2019; Cisneros et al., 2021; Cuberos-Urbano et al., 2018; Glazer et al., 2022) or individual programmes (Addy, 2011; Gehring et al., 2009; Rauwenhoff et al., 2022; Urech et al., 2020). Addy (2011) found a positive impact of psychoeducation, whilst others discussed delivering cognitive rehabilitation (Chalmers et al., 2019; Cisneros et al., 2021; Urech et al., 2020) and emotional coping strategies (Bay & Chan, 2019; Glazer et al., 2022; Urech et al., 2020). The cognitive rehabilitation studies included cognitive enrichment (Cisneros et al., 2021), problem-solving (Chalmers et al., 2019; Urech et al., 2020), goal management training (Cuberos-Urbano et al., 2018), direct attention training (Azulay et al., 2013), time management skills (Gehring et al., 2009), and self-awareness training (Cisneros et al., 2021).

The outcome of introducing strategies that created feelings of mastery was illustrated by improved anxiety and depression scores (Bay & Chan, 2019; Cisneros et al., 2021; Rauwenhoff et al., 2022; Urech et al., 2020) and by reports of enhanced physical, cognitive and emotional symptom management (Cuberos-Urbano et al., 2018). The context of strategy education appears to increase self-efficacy scores (Azulay et al., 2013) and QOL related to autonomy (Addy, 2011; Cuberos-Urbano et al., 2018; Glazer et al., 2022) in most studies. However, despite reporting positive changes in cognitive symptoms, Gehring et al.’s (2009), RCT including psychoeducation, computer-based rehabilitation and telephone booster sessions, did not find any significant changes in quality of life. There was therefore some element of conflict or uncertainty in this CMOC.

CMOC 4: If the intervention context includes others, a sense of connection is created, leading to reduced isolation and increased wellbeing

The ability to organize interventions to include people with ABI interacting with each other (Cisneros et al., 2021; Cuberos-Urbano et al., 2018; Glazer et al., 2022), or with their support systems, appears to activate a sense of connection (Cuberos-Urbano et al., 2018). This included group settings (Cuberos-Urbano et al., 2018), couple-based therapy (Milbury et al., 2020), and partners joining sessions (Rauwenhoff et al., 2022). Three studies discussed this sense of connection to others via group intervention (Cisneros et al., 2021; Cuberos-Urbano et al., 2018; Glazer et al., 2022). One study reported that a couples intervention promoted connection through a shared experience, reflection and discussion (Milbury et al., 2020) and a single case study pointed to the impact of including partners in sessions to encourage sharing of feelings (Rauwenhoff et al., 2022). The connection mechanism was also described as sharing and support (Cisneros et al., 2021), spontaneous peer support (Cuberos-Urbano et al., 2018), and stronger social ties and cohesion (Cuberos-Urbano et al., 2018).

When feelings of “connection” were activated, Rauwenhoff et al. (2022) reported a medium to large improvement in anxiety and depression scores, and Milbury et al. (2020) similarly reported reduced depressive symptomology. Studies also reported reduced isolation and loneliness (Cuberos-Urbano et al., 2018; Glazer et al., 2022), and reduced avoidance behaviour (Rauwenhoff et al., 2022). Interestingly, this mechanism was identified from the only online delivered intervention (Milbury et al., 2020), illustrating that even within an online format a sense of connection could also be promoted.

CMOC 5: If the intervention considers cognitive and psychosocial factors of the target population, then it is adapted, and this will meet the needs of the population and lead to better outcomes

Challenges experienced by people with ABI were often addressed by interventions that used adaptation as a mechanism, tailoring interventions towards the target population (Ashman et al., 2014; Bradbury et al., 2008; Chalmers et al., 2019; Gehring et al., 2009; Majumdar & Morris, 2019; Moustgaard, 2005; Rasquin et al., 2009; Thomas et al., 2019; Wathugala et al., 2019). Consideration of cognitive and psychological factors might include the type of ABI (Bomyea et al., 2017), the cognitive impact of an injury (Moustgaard, 2005; Roche, 2020; Wathugala et al., 2019), and/or the education background of the participant. When these contexts were considered, adaptation was used to initiate helpful responses. For example, authors described simplifying spoken and written language, using stroke-specific examples, reducing contrasting colour, shorter sessions, giving additional time to process learning, and prompts to read and practice the intervention.

Where adaptation was present, studies indicated a significant within-group decrease in depressive symptoms (Urech et al., 2020) and large quality of life improvements compared to an alternative (Cuberos-Urbano et al., 2018). Conversely, where studies did not use adaptations specific to the population, participants were not able to begin (e.g., when reminders were not offered, Ashman et al., 2014) or engage in (Rasquin et al., 2009; Wathugala et al., 2019) the intervention. Wathugala et al. (2019) reflected that a limitation of their intervention was not understanding the impact of cognition on participant ability to engage in varied mindfulness practice, and Rasquin et al. (2009) reported that only part of the sample improved on pre – and post-depression measures due to not all participants being screened for insight into their difficulties. Authors also reported that participants were unaware that the intervention being delivered could be relevant to their condition (Bomyea et al., 2017), and two studies reported improved QOL and symptom management, even when social and educational factors were not explicitly considered (Ashman et al., 2014; Bomyea et al., 2017).

CMOC 6: If a facilitator is responsive, then the intervention will be individualised, and the impact will be optimised for participants

Individualization of intervention was indicated as a mechanism activated in the context of a responsive facilitator. Studies outlined distinct ways to nurture this context. Studies proposed content-based adaptations, for example instructions for facilitators to encourage or prompt personalization, or personalizing treatment by tailoring intervention to individual or group goals or therapy targets (Potter et al., 2016; Urech et al., 2020; van Eeden et al., 2015). Exner et al. (2021) reported designing the intervention in a way that allows facilitators to select the intervention focus each session. Where individualization was used in this way, high participant satisfaction (Exner et al., 2021) and improved QOL were reported (Potter et al., 2016).

An alternative to the above was indicated by two studies. Urech et al. (2020) and Cuberos-Urbano et al. (2018) indicated that experienced therapists bring knowledge and skill to intervention delivery, creating helpful “spillover” effects and a space where individualization of the intervention is possible (Cuberos-Urbano et al., 2018; Urech et al., 2020).

### Phase 2: stakeholder consultation

Evidence from the stakeholder consultation was used to confirm, refute or contribute further understanding to how and why NPR programmes targeting PWB and/or QOL may or may not work. Provisional mechanisms determined by the literature were presented and a summary of the discussions are narratively described.

The group substantiated the importance of providing a space to learn and practice strategies (CMOC3), and this was extended with the suggestion that previous participants could act as co-facilitators in future groups (linking in with CMOC2 self-worth). The environment was deemed important, particularly creating connection and psychological safety within an online environment (CMOC4). Discussion on CMOC4 also extended to social-emotional functioning, considering how the intervention delivered to a group of patients could be extended to support relational frameworks. Regarding intervention delivery, the role of the facilitator/ therapist was discussed with the view that either the manual (for the future NPR intervention) needed to provide sufficient support, or the facilitator required skill in navigating process and complex interactions. A suggestion was made regarding the possibility of different facilitators for different topics.

### Phase 3: logic model

**Figure 2.**
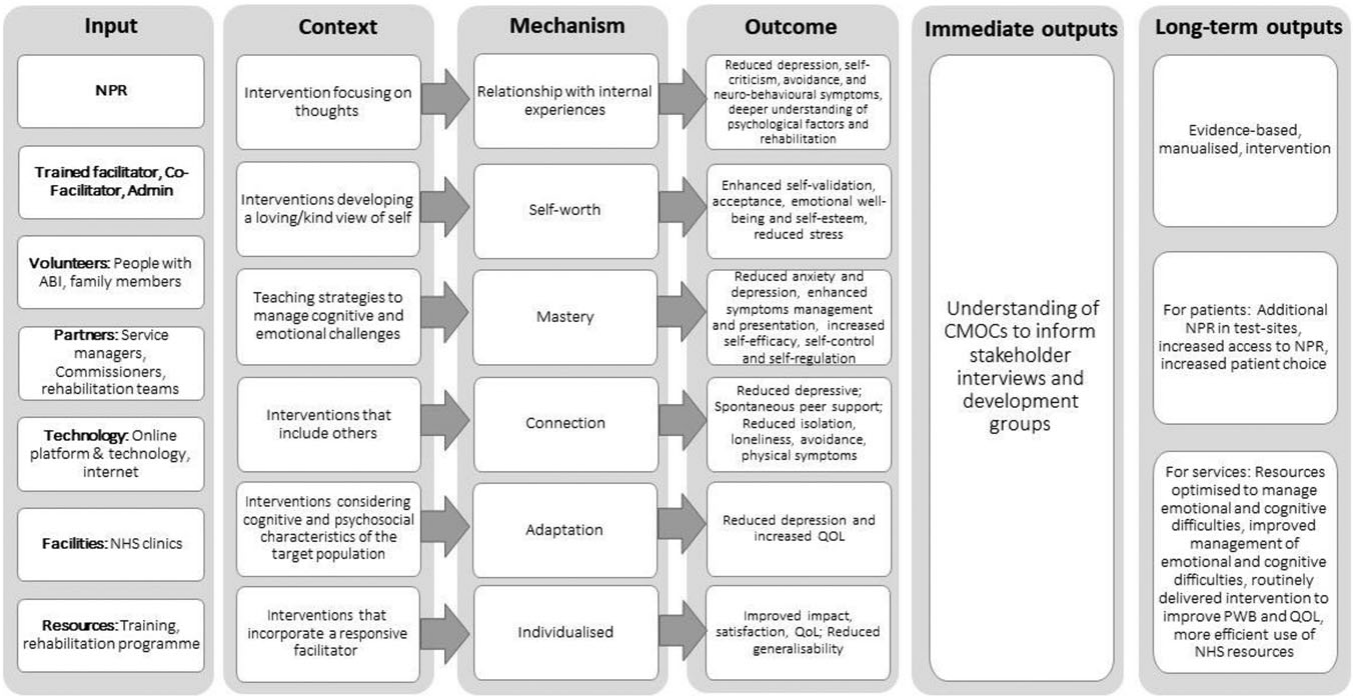
Preliminary logic model.

Synthesis of data from the literature search and stakeholder consultation was used to build a preliminary logic model (Figure 2).

## Discussion

We identified six key CMOCs (Table 3) related to positive outcomes following NPR for people with ABI. The participants’ relationships to their internal experiences, as well as feelings of self-worth, mastery, and connection appeared to be mechanisms that led to improved PWB and QOL. Adaptation and individualized programmes were also key mechanisms to explain how NPR programmes might lead to positive outcomes. These explanatory chains were then incorporated into the logic model (Figure 2) following stakeholder consultation.

## Comparison to the literature

Interventions that support participants to view their internal experiences from a different perspective, (CMOC1), enhance feelings of self-worth or compassion (CMOC2), and feel they have a sense of mastery over helpful strategies (CMOC3) are consistent with an NPR approach, which focuses on supporting people to return to participation in meaningful activities, despite their post-ABI cognitive, emotional, behavioural and functional challenges. Comprehensive and/or holistic NPR programmes are recommended for people after TBI in the post-acute setting (SIGN, 2013), with one systematic review pointing to the value of this approach to minimize the impact of moderate to severe injury (Cicerone et al., 2011). As well as cognitive rehabilitation programmes discussed by Cicerone et al. (2011), the National Institute for Clinical Excellence Quality Guideline (2014) also recommend “neuropsychological therapy” (p. 42). Therapies building traction in this domain include CBT, where systematic reviews indicate a positive impact on conditions such as anxiety (e.g., Little et al., 2021), anger (Iruthayarajah et al., 2018), and sleep quality and pain (Li et al., 2021), which is also confirmed by meta-reviews (Fordham et al., 2021). Although the evidence-base for third-wave psychotherapies (e.g., CFT, ACT) remains in its’ infancy, with methodological and conceptual issues highlighted, a recent systematic review indicates promise in this field (Robinson et al., 2019). Additionally, work is also underway to combine emotional and cognitive interventions into a neuropsychological psychotherapy group, and a recent single-case experimental evaluation (Sathananthan et al., 2022) has reported reliable improvements in emotional wellbeing and anxiety when taking this approach.

Consistent with literature indicating that loneliness can predict PWB and QOL (Salas et al., 2021), and with the renewed focus on supporting people with brain injury to develop and maintain stable and healthy relationships (e.g., Bowen et al., 2010; Riley et al., 2020), the current review highlights “connection” (CMOC4) as an important mechanism. Consistent with previous literature (das Nair & Lincoln, 2013; Withiel et al., 2019), the current review supports positive outcomes achieved when “connection” is activated through in-person settings. However, Whitten and Love (2005) report that participants anticipate reduced connection or rapport with the facilitator when interventions are delivered online. Although no online, group-based programmes have been evaluated following ABI, a recent qualitative study of an individual online memory intervention by Lawson et al. (2022) reported that more than half of clinicians and participants did not perceive any challenges to rapport building via telehealth. Of this sample, some reported challenges, such as informality resulting in reduced patient engagement, reduced reliance on body language and other non-verbal cues. However, quality of rapport was a dominant theme, with advantages (e.g., online settings feeling more relaxed and informal, being more integrated with participants home and family, promoting generalization of strategies) consistent with a proposed positive shift in power in online interventions, precipitated by both the professional and participant being in control of their own space (Jerome & Zaylor, 2000). Indication of positive outcomes is also consistent with physical rehabilitation programmes delivered online, where reduced isolation and social well-being were reported as benefits (Chen et al., 2019).

To facilitate participant engagement in NPR, the mechanisms of adaptation (CMOC5) and individualization (CMOC6) have also been identified. Adapting intervention to account for cognitive, cultural and social context is supported by the broader ABI literature. Simms et al. (2011) report the importance of selecting participants on a case-by-case basis, considering factors such as residual coping skills and cognitive challenges to engagement, prior to placing them in an online or group environment, and others advocate booster sessions for people undergoing interventions following ABI (Wong et al., 2018). Studies vary in reports of responsiveness to CBT following ABI. For example, Ponsford et al. (2016) indicated that higher premorbid intellectual functioning is associated with greater CBT benefits, and Hsieh et al. (2012) reported injury severity and severity of memory impairment as a modest predictor of poorer response. Fann et al. (2015) did not report interaction of injury severity or cognitive level on treatment response, and a study by Ponsford et al. (2020) reported that injury severity did not impact response to CBT when modified to need. Modifications for psychotherapy, and specifically CBT, have been advocated in systematic reviews (e.g., Gallagher et al., 2019), and include general (e.g., repetition and review, promoting continuity, use of concrete and personal examples, turning open questions in to multi-choice questions, handouts) and specific (e.g., recorded relaxations, short and tailored audio prompts) adaptations.

The recognized importance of the individualization mechanism (CMOC 6) is consistent with the broader evidence base. For example, individualized formulation-based approaches are promoted as central to implementation of psychological interventions broadly (Johnstone & Dallos, 2013) and after ABI (e.g., Evans, 2009). Promotion of individualization, flexibility and responsiveness is shared by biomedical personalized medicine and holistic person-centred care approaches (El-Alti et al., 2019). Lawson et al. (2022) emphasized the importance of flexibility and tailoring of interventions for people after stroke, suggesting that when this is done on an individual level, this is strongly linked to satisfaction. Research highlighted in our review also indicated facilitator skills, and literature has emphasized that individualization via facilitator scaffolding (i.e., giving the appropriate amount of support; Cuberos-Urbano et al., 2018; Urech et al., 2020) and/or helping participants to generate relevant examples can be beneficial, although this relies on facilitator experience and/or training.

## Strengths and limitations

This was a rapid realist review and therefore despite its success in formulating a preliminary logic model, has some limitations. Completing the review rapidly, to ensure timely contributions to the next stage of intervention development, may have made it more vulnerable to bias and limited its breadth and depth (Grant & Booth, 2009). However, as per realist review methods, and to avoid potential pitfalls of accelerated reviews (e.g., Moon et al., 2021), relevance and rigour appraisals were conducted on all the included studies. The lack of a traditional quality appraisal and subjective nature of the method could have introduced bias into data extraction and conclusions. Nevertheless, the method was conducted in line with other published realist reviews and a systematic approach was maintained throughout. Although possible, no studies were excluded based on relevance or rigour, and the findings were useful to support the confidence we placed on elements of the CMOCs, which were further confirmed and endorsed by the stakeholder consultation.

Furthermore, as this review was the first stage of a series of studies to be developed from the review in the process of complex intervention development, we took a pragmatic decision to ensure the review was completed to the pre-established timeframe and facilitated the next steps. It is therefore possible that additional useful material, and possible additional mechanisms, were not included. For example, the review could have focused on online, group-based interventions outside of the field of NPR (e.g., mental health). It could be argued that this may have provided additional and/or more relevant insights into the development of the new intervention, however the complexity of ABI presentations (e.g., cognitive, emotional and behaviour sequalae), and evidence suggesting adaptation of psychotherapeutic interventions in this population (e.g., Gallagher et al., 2019), influenced the authors decision to prioritise learning from current NPR programmes as the focus of this review. One way this learning could have been enhanced would have been to provide further detail on participant presentations in the data presented (e.g., aetiology, time since injury, cognitive impairment). Although the review had scope to be further expanded, and further details could have provided a greater understanding, we are relatively confident that the key CMOCs were identified, especially because these were endorsed by the stakeholder group.

The realist review was also constrained by the evidence-base itself. Prior knowledge of the paucity of evidence in this field supported the decision to use a realist review methodology focused on NPR delivered in ABI populations, in that, by using this methodology, the research team were able to use the limited information constructively by pulling together relevant information from disparate papers. However, the team accept that the results are only as good as the evidence available. Within the evidence base for NPR, very few authors explicitly mention mechanisms or explain “why” the interventions they report may or may not “work”. As realist methods aim to build explanation with the intention of advancing knowledge, the approach was deemed appropriate, but can only use the material that is available. This lack of detail is further supported by the incomplete TIDieR table from some of the included studies. This corroborates the difficulties experienced in extracting CMOC data and supports the strength of this review in producing useful material. A potential improvement may have been to the current review. Although TIDieR was selected as a checklist to understand intervention design for this review, the Rehabilitation Treatment Specification System (RTSS; Van Stan et al., 2018) may have delivered a theoretical framework designed to link hypothesized mechanisms with their treatment targets, possibly adding value to the outcome of the current review.

On a more conceptual level, the realist review focuses on studies that investigate how NPR impacts PWB and/or QOL independently in ABI. Definitions of PWB and QOL are broad, leading to a wide range of outcome measures considered as eligible. Such outcome measures focus on measuring either negative or positive affect respectively. By focusing only on outcomes that measure affect extremes, and by accepting that some studies included may have selected only one extreme to measure, the review may have inadvertently missed an opportunity to learn about rehabilitation intervention that simultaneously targets negative and positive affect disturbances, which are thought to lead to improved functioning, and long-term recovery (e.g., Fried et al., 2016).

Finally, considering the complexity of the intervention, it could be argued that the logic model developed is an oversimplified illustration. This critique has been reported in other research using this method (e.g., Hinds & Dickson, 2021). However, as the first realist review in NPR completed by a clinically and methodological experienced research team, the findings provide a vehicle to begin communicating the complexity of these interventions in a way that is theoretically recognized and plausible. Also, the current project framework is following the complex intervention development guidelines (O'Cathain et al., 2019; Skivington et al., 2021) and will include continued further exploration and explanation of the findings through stakeholder involvement and preliminary testing.

## Relevance

Our review has provided researchers and clinicians with a comprehensive framework for understanding the contexts and mechanisms of NPR that relate to positive PWB and QoL outcomes for people with ABI. This framework will be further refined and tested in the ongoing project before it is considered ready for implementation in a clinical setting.

Although relevance of studies identified was satisfactory for the purpose of this review and the ongoing project, there is a gap in the NPR evidence-base for individual and group online delivery formats, for NPR delivered to people after ABI diagnoses other than stroke and/or TBI, and for outcomes beyond those focusing directly on the patient context. For this reason, ongoing intervention development will require work focused on engaging with professional groups, for example healthcare and IT professionals, and with patient stakeholders to gather additional views. Outcome measures focussing on relational systems should also be considered.

## Future research

The review has indicated a need for NPR interventions to be reported using the TIDieR (Cotterill et al., 2018) checklist. The review also points to the requirement for consistency in outcome measurement in NPR, and possibly to include outcome measures that explore the experience beyond the immediate participant (e.g., carer experience).

Whist the literature suggested that the NPR interventions identified have a positive impact on the outcomes described for people after ABI, more work is needed to build and establish the quality of the evidence-base. NPR identified for this review was dominated by face-to-face/in-person delivery formats and delivered to those with stroke and/or TBI. Future research should seek to expand the evidence for alternative delivery formats (e.g., online delivery), and build evidence in broader diagnostic categories (e.g., hypoxia, encephalitis). Although beyond the scope of this review, evidence was available for NPR delivered to or by relational systems, for example the neurological rehabilitation team and/or family, as well as for NPR where PWB and/or QOL were secondary outcomes. It is likely that a review of this literature would strengthen and develop the logic model created here.

## Conclusions

The logic model developed and refined from this review can explain and improve how NPR interventions are configured for ABI survivors. The review highlights mechanisms that maximise the success of NPR programmes and offers key contexts in which these mechanisms are most likely to be activated. Specifically, contexts need to be considered that promote the six mechanisms outlined. We believe this review offers a timely contribution to the existing literature and will be used to refine and test a new NPR intervention for ABI survivors.

## Supplementary Material

Supplementary File 1.docx

Supplementary File 3.docx

Supplementary File 2.docx
