# Supplementary material for: A rapid realist review of clinical neuropsychology rehabilitation programmes to improve psychological wellbeing and quality of life for people with acquired brain injuries
Source: Neuropsychol Rehabil. 2023 Nov 17;34(8):1035–70. doi: 10.1080/09602011.2023.2273580 (PMC11332407; doi:10.1080/09602011.2023.2273580)
Supplement: Supplementary File 3.docx [file PNRH_A_2273580_SM8930.docx]

| STUDY NUMBER | authors | Why (Aim) | What | | Who provided (Facilitator) | How | | Where (Location/infrastructure) | When and how much | | | Tailoring | Modifications | How well | | |
| --- | --- | --- | --- | --- | --- | --- | --- | --- | --- | --- | --- | --- | --- | --- | --- | --- |
|  |  |  | **MATERIALS** | **PROCEDURES** |  | **MODE OF DELIVERY** | **DELIVERY FORMAT** |  | **NUMBER OF SESSIONS** | **SESSION LENGTH** | **SESSION FREQUENCY** |  |  | **LIMITATIONS** | **RELEVANCE** | **RIGOUR** |
| 1 | ADDY (2011) | To gradually increase activity while addressing unhelpful cognitive interpretations of symptoms | Copy of assessment results in a formal report; activity record; | Psychoeducation, pacing program, reflective practice | Clinical Neuropsychologist | F2F | Individual | - | 6 | - | Monthly | Tailored to the specific needs and expectations of the individual, focused on managing symptoms rather than eliminating the symptoms | - | - | 2 | 3 |
| 2 | ASHMAN ET AL. (2014) | To examine the effectiveness of CBT and SPT in reducing depressive symptoms in people with TBI who met criteria for a depressive disorder. | CBT: Memory supports; written handouts; diary; relaxation tape and written description | CBT: cognitive restructuring techniques; guided self-dialogue techniques; mastery of planning techniques; homework/practice   SPT: client-cantered approach; naming the problem; praise; reassurance; encouragement; psychoeducation and advice; anticipatory guidance; expanding awareness | Postdoctoral fellows in clinical neuropsychology and rehabilitation psychology with at least 2 years of previous supervised experience conducting psychotherapy and at least 1 year of experience working with individuals with TBI | F2F | Individual | - | 16 | Initial session - 90 minutes; subsequent sessions - 50 minutes | Twice weekly for the first month and then weekly (3 months in total) | CBT: adapted to address impairments specific to people with TBI by embedding training of compensatory strategies within treatment sessions such as memory supports and organisational strategies | For participants who could not attend sessions more than once a week, treatment was delivered once weekly over 4 months | 1. Sample size smaller than intended   2. Heterogenous sample in terms of severity of injury and time since injury   3. High preintervention dropout rate   4. Occasional non-adherence to standardised treatment protocol | 5 | 4 |
| 3 | ASHWORTH ET AL. (2011) | To integrate CFT into CBT as CBT alone was not effective treatment for the patient | Visual diagrams | Thoughts records, psychoeducation, diary of self-critical thoughts | Clinical psychologist | F2F | Individual | Neurorehabilitation centre | 24 | 50 minutes | Weekly | Personalised to individual formulation and goals (increasing and self-esteem)  50-minute sessions, rather than 1-hour; short break if necessary | - | 1. Single case, but not single case experimental design methodology  2. Intervention as part of holistic rehabilitation programme, so cannot assume changes are solely due to CFT  3. No long-term follow-up | 5 | 1 |
| 4 | AZULAY ET AL. (2013) | To evaluate the effectiveness of the mindfulness-based stress reduction (MBSR) program tailored to  individuals with mild traumatic brain injury (mTBI) as patients with mTBI tend to experience increased endorsement of symptoms and reduced self-efficacy compared to more severe injuries. | Manual, homework log, assignments provided in writing | Consistent with Kabat-Zinn MBSR programme | 2 Neuropsychologists | F2F | Group | Post-acute brain injury rehabilitation centre within suburban rehabilitation hospital | 10 | 120 minutes | Weekly | Group size reduced from 25 to an average of 6 participants; increased number of sessions from 8 to 10 to allow time required to explain concepts, repeat procedures and reinforce learning process; increased amount of modelling of more sophisticated techniques such as using mindfulness for exploration of emotional and physical pain | - | 1. All participants were receiving concurrent rehabilitation, so impossible to isolate the effects of MBSR  2. No control group or randomisation to treatment  3. Relatively small sample size  4. No specific measures of emotional regulation and mental health | 5 | 3 |
| 5 | BAY & CHAN (2019) | To evaluate whether an 8-week mindfulness-based group therapy compared to health promotion active control group therapy reduces chronic stress, TBI symptoms, and depressive symptoms; self-management theory and contemplative science | Written summaries and recordings of the sessions for individuals who could not attend | PFM-GT: positive body awareness, mindful movement (as well as mantra), mindful and compassionate meditation;  AC-GT: health promotion to improve knowledge and skills on preserving brain health and reducing stress, evidence-based practices with exercise, nutrition, and sleep hygiene | Trained facilitators with mental health and/or mindfulness backgrounds | Mixed (F2F + phone) | Group | - | 4 F2F + 4 phone-based booster classes | 90 minutes (F2F); 60 minutes (phone) | - | The current intervention challenged the in-person traditional format by allocating less time (360 minutes) to in-person class time than traditional mindfulness interventions and providing booster telephone classes (240 minutes) to develop use of mindfulness skills in daily life while reducing the burden of travel to attend class | - | 1. Sample mostly in suburban setting and associated with a university - not generalisable to other communities;  2. Data collected by self-report - subject to recall bias;  3. Home practice could not be determined with the daily log as only eight individuals returned their final log;  4. Randomisation was not possible for all due to life circumstances, including completing medical appointments, childcare responsibilities, and family commitments;  5. Although researchers provided a recording and paper copies of the class activities to absent individuals, the strength of these substitutions to the outcomes is unknown | 5 | 4 |
| 6 | BOMYEA ET AL. (2017) | To describe psychotherapy response in veterans with and without TBI | ACT and PCT manuals | ACT: brief therapy rationale, mindfulness exercise, homework review, discussion of a new topic, metaphors, experiential activities, new homework assignment  PCT: psychoeducation, identification of daily life stressor, insight-oriented and supportive techniques | Therapist | F2F | Individual | - | 12 | 60 minutes | Weekly | The ACT treatment manual was designed for use in a transdiagnostic veteran population | - | 1. Self-report history of head injury - memory and reporting bias  2. Limited data on the severity of the brain injury  3. No assessment of compensation status | 4 | 4 |
| 7 | BRADBURY ET AL. (2017) | To evaluate the efficacy of cognitive behaviour therapy (CBT), adapted to meet the unique needs of individuals with acquired brain injury (ABI), and modified for both group and telephone delivery. | - | CBT; education and information provision | CBT: 2 clinical psychologists with 5 years of CBT delivery, co-facilitator - master's-level graduate student  Education control: master's-level graduate student | F2F OR phone-based | Group OR Individual | - | 11 (1 introductory session, 10 treatment sessions) | - | Weekly or twice weekly (9 weeks in total) | Specific cognitive information about each patient was obtained from neuropsychological assessment  results. Specifically, the presence and severity of current cognitive deficits were identified in the following areas: memory, attention, initiation/activation, language, and speed of processing. For each patient, (1) their ability to benefit from repetition, (2) their capacity to learn and retain information, and (3) speed of information processing were identified and provided the therapist with information that helped determine the rate and  complexity of speech and amount of repetition and summarization that might be required to optimize retention of information for each patient. | - | 1. An education-matched study, not a randomized controlled trial  2. Small sample size  3. Representativeness of the sample - all individuals who participated in the current project were connected to a community ABI program and lived in urban areas with access to high-quality health care  4. Sample bias | 5 | 4 |
| 8 | CHALMERS ET AL. (2019) | To investigate the efficacy of problem-solving therapy for reducing the emotional distress experienced by younger stroke survivors. | - | Problem solving therapy explanation and rationale, problem definition, goal setting, solution generation, solution selection, and action plan implementation | - | F2F | Group | - | 6 | 90 minutes (first 2 sessions); 60 minutes | - | The first six steps (problem solving therapy explanation and rationale, problem definition, goal setting, solution generation, solution selection, and action plan implementation) were spread over the first two sessions to allow for longer time required to learn each step in a group setting | - | 1. No randomisation due to patients' localities - potential selection bias;  2. Potential confounding variables: therapist attention, social support, placebo effects;  3. Passing of time as possible explanation of difference between baseline and follow-up  4. Does not account for needs of younger people.  5. Broader age inclusion criteria than intended  6. Participants included if reported anxiety/depressive symptoms but no formal diagnosis  7. Coping was not measured.  8. Lack of improvement in problem-solving may be explained by baseline normal scores on problem-solving measure | 4 | 4 |
| 9 | CISNEROS ET AL. (2021) | To evaluate the impact of a 12-week, 24-session multimodal group cognitive  intervention, the Cognitive Enrichment Program (CEP), on episodic memory in older adults with traumatic  brain injury (TBI) compared to an active control group that received usual care in the form of individual holistic rehabilitation. | - | Introduction and self-  awareness, Attention and memory, and Executive functions; homework included reading magazines or newspaper articles to practice the SCT method, meeting at least one new person during the week to practice the face-name method, and making one grocery list and one to-do list during the weekend. | Clinical Neuropsychologist | F2F | Individual | Rehabilitation centre | 24 | 90 minutes | Twice weekly | Provided in supplementary materials | - | 1. It is impossible to determine whether only the memory module of the CEP intervention is related to the observed  changes in the experimental group.  2. The small size or their sample does limit the scope of our conclusions.  3. Some effects of CEP interventions may have been masked by the positive influence of the holistic rehabilitation approach (standard car-confounding variable) | 4 | 4 |
| 10 | CUBEROS-URBANO ET AL. (2018) | To identify the potential target and effect size of goal management training (GMT) enhanced with life-logging technology compared with standard GMT on a range of possible primary outcomes reflecting cognitive and ecological aspects of executive functioning and quality of life. | Trainer's manual, presentation, homework materials, lifelog recordings (SenseCam and ActiHeart) | As described in Levine et al. (2011); physical rehabilitation and psychoeducation | Therapists (authors) | F2F | Group | Rehabilitation Unit at a hospital | 14 | 60 minutes | Twice weekly | Lifelog recordings were used for three main purposes: (1) to identify everyday situations in which goal-neglect behaviours arise; (2) to provide specific feedback about these real-life problems via standard GMT strategies; and (3) to raise awareness and boost ongoing monitoring of slips in subsequent everyday situations (between sessions). | - | 1. Use of questionnaires and self-reports - possible subjective bias  2. Pilot 'proof of concept' study  3. Too small sample size to detect a significant effect and draw conclusions about the comparative efficacy of these two intervention modalities | 3 | 3 |
| 11 | DI VITA ET AL. (2022) | To explore the effect of Psychotherapy and Art Therapy on different psychological aspects in patients with a diagnosis of severe Traumatic Brain Injury (TBI), a subset of the larger ABI group, who were at least 12 months post-injury and had completed the traditional rehabilitation program. | Provided in supplementary materials | Provided in supplementary materials | - | F2F | Group | - | - | - | 4 months (psychotherapy) + 4 months (art therapy) | - | - | 1. Small sample size - heterogeneity affected in clinical and demographical variables | 3 | 2 |
| 12 | EXNER ET AL. (2021) | To investigate the treatment outcome of an integrated intervention, combining neuropsychological and cognitive behavioural therapy (nCBT), against waitlist (WL) in outpatients with ABI. | Manual, written treatment materials and session summaries, recapitulations of topics covered, and prompts and reminders to facilitate the completion of homework | Cognitive remediation, cognitive behavioural strategies, neurorehabilitation, problem-solving framework, six manualized treatment modules, five-step problem-solving model, problem-solving strategies, psychoeducation, compensatory strategies, CBT framework, cognitive reappraisal techniques, supportive appraisal of  present achievements, using self-reinforcement and reducing dysfunctional attitudes towards improvements, such as comparisons with previous performance levels and perfectionism, cognitive and emotional stimulation | Five trainee clinical psychologists, 2 licensed clinical psychologists with training in both neuropsychological therapy and cognitive–behavioural therapy | F2F | Individual | - | <80 | 50 minutes | Weekly | Duration of treatment tailored to the individual patient’s needs + materials | - | 1. No formal evaluation of treatment fidelity in terms of monitoring the therapists’ compliance with the manual or the therapists’ competency was carried out  2. The design of the study makes it impossible to name specific therapeutic strategies that will be most  effective for addressing specific cognitive or emotional deficits  3. Compared to routine clinical care, our trial provided more support for therapists in terms of the semi-standardized manual and regular supervision. Because of this, the extent of the observed effects may not be completely transferable into routine care  4. Very cost-intensive approach, which may not be transferable to many other health care systems with more limited resources | 5 | 4 |
| 13 | GEHRING ET AL. (2009) | To evaluate the effects of a multifaceted cognitive rehabilitation program (CRP) on cognitive functioning and selected quality-of-life domains in patients with gliomas who often experience cognitive deficits, including problems with attention and memory | Computer program (C-Car); homework | Computer-based attention retraining exercises, logs kept about experiences with applying compensatory strategies in daily life; a series of hierarchically graded tasks designed to strengthen various aspects of attention on the basis of patient needs; six psychoeducation sessions that addressed attention, memory, and executive function;  telephone-based booster session, during which key aspects of the compensation training were re-emphasized | Neuropsychologist | Mixed (F2F + computer-based) | Individual | - | 6 | 120 minutes | Weekly | - | - | 1. Practice effect or regression to the mean could have initially overwhelmed the intervention effects.  2. Large number of outcome measures.  3. Study results can only be generalized to glioma patients who both report having cognitive symptoms and score below a predetermined cut-off on objective NP tests  4. The study could not tease out the relative effectiveness of cognitive retraining versus the use of compensatory strategies | 4 | 4 |
| 14 | GERTLER & TATE (2021) | To investigate whether BA improves activity participation and mood for people with depression following brain injury | Revised treatment manual by Lejuez et al. (2011) | Scheduled activities outside intervention at home or in the community | Registered psychologist | F2F | Individual | Patient’s home | 10-14 | 30-90 minutes | Weekly | - | - | 1. Participants expressed the intervention did not meet their needs. | 3 | 3 |
| 15 | GLAZER ET AL. (2021) | To inform the development of a cognitive rehabilitation and psycho-social group intervention for Teenage and Young Adult (TYA) survivors of brain tumours | Workbooks, visual aids | personalising of workbooks, cognitive strategies, identifying identities and personal qualities, impact of brain tumour, peer interaction, strategies for managing social and emotional effects, goal setting | 2 clinical psychologist and an assistant psychologist | F2F | Group | Outpatient oncology service | 1 | 1 day | - | Specifically developed intervention | - | - | 4 | 4 |
| 16 | HART ET AL. (2020) | To assess whether brief BA, followed by SMS-delivered implementation intentions, would result in improved outcomes compared to a control treatment session on the importance of motivation followed by SMS-delivered motivational messages | Manual, trouble-shooting guide, text messages, message schedule | Lay-language education, a list of possible activities to engage in during the trial,  8–10 implementation intentions,  action plans, coping plans | therapists | Mixed (F2F + text messages) | Individual | - | 1 | - | - | The number of text messages and specific messages where chosen by participants in both conditions | - | 1. No measure of clinical significance.  2. Relatively small sample  3. Participants with mild depression/anxiety were included - possibly limiting the range of improvement | 4 | 4 |
| 17 | HOFFMANN ET AL. (2015) | To evaluate the efficacy of a self-management intervention and a coping skills intervention, compared to usual care, on anxiety and depression post-stroke. | Additional checklist titled ‘‘Identifying areas of concern’’; 'What you need to know about stroke’ education package checklist and subsequent provision of a tailored written education package; a structured plan using a goal setting aid called "My Action Plan"; | Self-management: relaxation/stress management techniques; cognitive and emotional education; fatigue management; goal setting; referral to community providers; education about stroke; return to driving support and advice; return to work advice; communicating with healthcare professionals; education about communication concerns; support for basic daily activities; | Health professionals - not stroke team, specifically hired to deliver the intervention | F2F | Individual | first 2 sessions in hospital before discharge, the rest of sessions in patient's home | 8 | 60 minutes | Weekly | Tailored to individual needs | Coping: 1 participant had all sessions in hospital; 1 participant had all sessions at home; self-management: 1 all home, 1 all hospital, 3 half/half | 1. Small sample size - possibility of Type II error  2. No qualitative component  3. Some outcome measures may not be sensitive enough to detect psychosocial changes | 4 | 4 |
| 18 | KOOTKER ET AL. (2017) | To evaluate the effectiveness of individually tailored cognitive behavioural therapy (CBT) for reducing depressive symptoms with or without anxiety poststroke. | - | Augmented CBT tailored to subject own activity-related goals or computerised (COGNIPLUS) - self-determined cognitive domains at own performance level | MDT (Health Psychologist, OT, Movement Therapist) | F2F | Individual | Rehabilitation institutes | 13-16 | 20-25 minutes (60 minutes with breaks) | Within 4 months | Augmented CBT tailored to goals; computerised, patient tailored to cognitive domains | - | 1. No care as usual control group  2. Small sample size, different power calculation  3. Requirement for larger sample of multiple sites  4. Missing values of T3  5. No follow up or modelling | 2 | 4 |
| 19 | MAJUMDAR & MORRIS (2019) | To evaluate the efficacy of group-based ACT for stroke survivors in comparison with treatment as usual (TAU) controls | Leaflets detailing the course content and aims (‘a short course to help you get on with your life after stroke’)  didactic PowerPoint group sessions | Week one: Creating the distinction between actions under our own control and actions controlled by our mind.  Week Two: Focus on acceptance (not resignation) and willingness to experience unpleasant feelings and sensations (e.g., pain or anxiety) without attempts to fight, avoid or suppress them. | Clinical psychologists, assistant psychologists/ stroke care coordinators. | F2F | Group | Community venues, for example, libraries | 4 | 120 minutes | Weekly | Reducing contrasting colours, simplifying the language and number of words on the slides, and inclusion of stroke-specific examples | - | 1. Relatively small sample which would not permit more sophisticated statistical analyses without compromising statistical power  2. Despite randomization, the control and intervention samples differed significantly in gender composition and showed a near significant difference for living arrangement  3. This study was unable to provide equality of therapy hours to compare against an active treatment or undertake independent checks for treatment adherence  4. The 2-month follow-up may not have been sufficient for such life changes to occur in response to the intervention | 4 | 5 |
| 20 | MILBURY ET AL. (2020) | To examine the feasibility and preliminary efficacy of a couple-based meditation (CBM) program targeting symptom and well-being outcomes | Values worksheet, homework worksheets | Cultivating mindfulness, compassion, gratitude and purpose, and integrated emotional disclosure exercises. | Master-level licensed psychological counsellor intern | Online | Couples | FaceTime (Apple Inc, Cupertino, CA) | 4 | 60 minutes | Weekly | - | - | 1. Sample has homogenous characteristics particularly regarding race/ethnicity and socioeconomic status  2. No active comparison arm for attention and support (group effects might be due to this)  RCT not powered to examine group differences, efficacy must be interpreted with caution | 4 | 3 |
| 21 | MITCHELL ET AL. (2009) | To evaluate long-term efficacy of  brief behavioural intervention, adjunctive to antidepressant therapy in people with poststroke depression | - | As described here: [Mitchell](https://doi.org/10.1016/j.jstrokecerebrovasdis.2007.12.002 Mitchell) et al., 2008 | Nurse | F2F | Individual | - | 9 | - | 8 weeks | Stroke-specific language, individualised approach | - | - | 5 | 5 |
| 22 | MOHAMAD ET AL. (2020) | To determine the effect of ACT interventions on changes in the quality of life of post-stroke patients at Aloe Saboe Hospital. | - | - | - | - | - | Hospital | - | - | - | - | - | - | 2 | 2 |
| 23 | MOUSTGAARD (2005) | 1. To adapt and implement Mindfulness Based Cognitive Therapy (MBCT) for stroke survivors;  2. To evaluate the psychometric properties of generic and stroke-specific measures;  3. To evaluate the effectiveness of MBCT on aspects of quality of life, emotional factors, and adjustment for participants, as well as caregiver burden  4. to determine the predictors of success | Homework, logbooks | Mindfulness meditation, introductory to more advanced aspects of cognitive therapy (in a group format), education about stroke and accompanying emotional changes, a weekly review of events and homework, and group work, introductory yoga techniques | Group leaders | F2F | Group | - | 9 | 105 minutes | Weekly | Accommodated the needs and issues in individuals with stroke |  | 1. No wait-list control group.  2. Small sample size.  3. Potential threats to internal validity.  4. Potential experimenter and subject bias. | 4 | 3 |
| 24 | OLIVE (2000) | To evaluate the efficacy of brief CBT in 5 stroke patients with PSD | Mood tracking questionnaire | Mood tracking | Author trained in CBT | F2F | Individual | - | 5-7 | 40-55 minutes | Thrice weekly | - | - | 1. Potential selection bias (only high-functioning individuals were selected)  2. Limited screening procedures  3. Ethnic bias (systemic)  4. Integrity of the manual was not checked  5. Author acted as a therapist - investigator bias  6. Many cases of PSD resolve spontaneously  7. Strict inclusion criteria  8. Patients received inpatient rehabilitation - expected improvement | 3 | 3 |
| 25 | OLUKOLADE & OSINOWO (2017) | To examine efficacy of cognitive rehabilitation therapy (CRT) in the treatment of PSD among stroke survivors | - | CRT: activity stimulation (3 sessions), negative thoughts (3 sessions), people contacts (3 sessions); PET: knowledge on stroke and poststroke depression; CG: usual care in hospital | - | F2F | Group | Hospital | 9 | - | Over 3.5 months period | - | - | 1. Small sample size | 4 | 4 |
| 26 | POTTER ET AL. (2016) | To evaluate the effectiveness of a 12-session individualised, formulation-based CBT programme | Homework, semi-structured protocol (copies of the protocol and booklets are available from the authors upon request) | Agenda-based sessions, collaborative target setting, problem identification, psychoeducation, socialising the  patient to the CBT model and formulation, individual target problems identified collaboratively with the therapist, relapse prevention, how to maintain therapeutic gains | Clinical neuropsychologist with previous experience of CBT in context of TBI, depression and chronic fatigue syndrome | F2F | Individual | - | 12 | 60 minutes | Weekly | Individualised | - | 1.  Variation in time taken to complete the sessions of CBT  2.  Cross-over waiting list design complicates the controlled assessment of the maintenance of therapy benefits and longer-term outcome  3. Data on other variables that might have influenced outcomes (such as homework completion, or perceptions of the usefulness of treatment) were not collected  4. Single therapist combined with individualised treatment - limited generalisability  5. Potential response bias  6. No measure of therapist competence and protocol adherence | 5 | 5 |
| 27 | RASQUIN ET AL. (2009) | To investigate whether or not a cognitive-behavioural intervention for depression after stroke has an effect and is feasible | Intervention book (theoretical background)  Diary to record mood and antecedent events  Homework | Planning pleasant activities, recognise negative thoughts, relaxation, pleasant activities, cognitive restructuring, relaxation exercises, recording mood | Psychologist | F2F | Individual | - | 8 | 60 minutes | Weekly | Psychology assistant contact 2 x per week to offer help | - | 1. Single case, limited generalisation  2. Could not randomise the baseline  3. Difficult to control for confounding effects (e.g., other therapies, medication use, impact of events)  4. Short follow-up  5. No control group | 4 | 2 |
| 28 | RASQUIN ET AL. (2010) | To help patients and caregivers to gain insight in to ABI consequences, to offer strategies to handle cognitive deficits, to learn social skills to live with consequences, to learn how to control emotional reactions, to enhance self-efficacy | - | Cognitive training focused on strategies (coping, anxiety, memory, etc.), social skills training, role-play, assertiveness training | 2 Cognitive therapists | F2F | Group | - | 15 | 150 minutes | Weekly | Each patient formulated individual strategies. For each cognitive domain, specific exercises were used in which the individual problems of the participants were taken into account | - | 1. Only an initial insight | 4 | 2 |
| 29 | RAUWENHOFF ET AL. (2022) | To investigate the effectiveness of Acceptance and Commitment Therapy for patients with ABI | BrainACT intervention protocol,  homework exercises in workbook | Reading or listening to session summaries, practicing skills, mindfulness | Psychologist trained in ACT | F2F | Individual | - | 8 | 90 minutes | Weekly (first 4 sessions) or biweekly | - | Modifications for possible cognitive deficits, response to brain injury topics discussed during treatment | 1. Scores interpreted with caution due to autocorrelation  2. Young and high education sample  3. Not independent treatment adherence rating (and pp 1 low)  4. No conclusions can be made from the study | 4 | 3 |
| 30 | ROCHE (2020) | To illustrate the management of Post-Traumatic Stress Disorder in the context of Traumatic Brain Injury (TBI), using an Acceptance and Commitment Therapy based approach | CALM app | Psychoeducation to the model - key values, patterns of TFB, maintenance of distress  Acceptance - relaxation, breathing exercises, positive self-talk  Commitment - behaviour exposure programme | Neuropsychologist | F2F | Individual | Patient’s home | 15 | 90 minutes | Every 2-3 weeks | Individualised intervention | - | 1. Small sample  2. No control group | 4 | 2 |
| 31 | THOMAS ET AL. (2019 | To increase activity, particularly frequency of pleasant or enjoyed activity, and to improve mood using Behavioural Activation | BEADS therapy manual | Activity monitoring, activity scheduling and graded tasks, identifying enjoyable activities. | Assistant Psychologist or psychological well-being practitioner | F2F | Individual | - | ≤15 | - | 4 months total | Tailored to individual needs | - | 1. Target recruitment was not achieved | 4 | 4 |
| 32 | URECH ET AL. (2020) | To understand if integration of neuropsychological and psychotherapeutic treatment is more effective than neuropsychological treatment alone in ABI in adjustment disorders | Manual | Individual treatment plan, psychoeducational rationale focused on problem solving and coping, interventions adapted from CBT, emotion-focused, process-experiential, interpersonal | 3 psychologists (neuropsychologist or psychotherapists) | F2F | Individual | - | <20 | - | <1 year total | Treatment length adapted to patients’ needs | - | 1. Small sample size (possibly broader inclusion criteria)  2. No measures of adherence for both therapies  3. Maybe two groups not dissimilar  4. Delivered by Neuropsychologist and CBT therapist = possible spill over effects  5. Heterogenous sample | 5 | 4 |
| 33 | VAN EEDEN ET AL. (2015) | Cost-effectiveness and cost-utility evaluation of a cognitive behavioural therapy augmented with occupational and movement therapy to support patients with a stroke with depressive symptoms in goal-setting and goal attainment (augmented CBT) in comparison with a computerized cognitive training program (CogniPlus) as a control intervention | Augmented CBT tailored to subject own activity-related goals  Computerised (COGNIPLUS) - self-determined cognitive domains at own performance level | 10-12 sessions, with Health Psychologist; 3 sessions with OT or Movement Therapist. If HADS >7, additional OT/MT session. | Health Psychologist, OT, MT | F2F | Individual | Rehabilitation centres, rehabilitation department in a hospital | 10-12 | 20-25 minutes (30-40 minutes with breaks) | Over a 4 months period | CBT tailored to goals | - | 1. No care as usual control group  2. Small sample size, different power calculation  3. Requirement for larger sample of multiple sites | 2 | 4 |
| 34 | WANG ET AL. (2020) | To investigate whether mindfulness meditation has potential benefits in PSD and QOL for sICH patients in China. | - | MCBT: learning to respond adaptively to thoughts, feelings, and experiences; Stress management education (SME - control): lectures covered topics relevant to stress, stress physiology, insomnia, exercise, and nutrition | MBCT therapists | F2F | Group | - | 8 | 120 minutes | Weekly | - | - | 1. Use of self-report measures of mindfulness  2. MBCT used as mindfulness-based intervention but it incorporates elements of CBT - unsure if effects due to mindfulness or CBT components  3. Non-representative sample  4. No follow-up | 5 | 5 |
| 35 | WATHUGALA ET AL. (2019) | To find low-cost, effective, and accessible methods to help spasticity and promote recovery | MP3 player, pre-recorded audio based on Jon Kabat-Zinn 8-week MBSR course | Body Scan Meditation, Sitting Meditation, working with difficulty meditation, Breathing Meditation, | Experimenter + self-led | Mixed (F2F + self-directed) | Individual | Patient's home | 14 | 30-40 minutes | Daily | Intro to mindfulness with experimenter present to ask questions; reduced 8-week course to 2-weeks (mirrored first 2 weeks of MBSR programme) | - | 1. Do not carefully examine the role of cognitive function (no objective measures) and this could have impacted meditation practice  2. Small sample  3. No control group | 4 | 2 |
