# Supplementary material for: A rapid realist review of clinical neuropsychology rehabilitation programmes to improve psychological wellbeing and quality of life for people with acquired brain injuries
Source: Neuropsychol Rehabil. 2023 Nov 17;34(8):1035–70. doi: 10.1080/09602011.2023.2273580 (PMC11332407; doi:10.1080/09602011.2023.2273580)
Supplement: Supplementary File 2.docx [file PNRH_A_2273580_SM8929.docx]

**Search Strategy**

| **Platform and database:** Ovid MEDLINE(R) ALL <1946 to March 28, 2022> **#** | **Search term(s)** |
| --- | --- |
| 1 | cerebrovascular disorders/ or exp basal ganglia cerebrovascular disease/ or exp brain ischemia/ or exp carotid artery diseases/ or stroke/ or exp brain infarction/ or exp cerebrovascular trauma/ or exp hypoxia-ischemia, brain/ or exp intracranial arterial diseases/ or exp "intracranial embolism and thrombosis"/ or exp intracranial hemorrhages/ or exp vertebral artery dissection/ |
| 2 | (stroke* or "post stroke*" or post-stroke* or "cerebral vascular" or cerebrovascular or cva*).tw,kw,kf. |
| 3 | ((cerebral or brain* or vertebrobasilar) adj3 (infarct* or isch?emi* or thrombo* or apoplexy or emboli*)).tw,kw,kf. |
| 4 | ((cerebral or brain or subarachnoid) adj3 (h?emorrhage or h?ematoma or bleed)).tw,kw,kf. |
| 5 | exp brain injuries, traumatic/ or brain injuries/ |
| 6 | ((trauma* or acquired) adj3 brain injur*).tw,kw,kf. |
| 7 | exp brain abscess/ or exp central nervous system infections/ or exp encephalitis/ or exp meningitis, viral/ |
| 8 | (encephalitis or meningitis).tw,kw,kf. |
| 9 | exp brain neoplasms/ |
| 10 | ((brain or cerebr*) adj3 (neoplasm* or lesion* or tumor* or tumour*)).tw,kw,kf. |
| 11 | exp hypoxia/ |
| 12 | (hypoxi* or "oxygen deficienc*").tw,kw,kf. |
| 13 | or/1-12 |
| 14 | (neuropsych* adj2 (intervention* or rehab* or psychotherap*)).tw,kw,kf. |
| 15 | ((cognitive or relational) adj2 rehab*).tw,kw,kf. |
| 16 | exp cognitive behavioral therapy/ or exp couples therapy/ or social cognition/ |
| 17 | ("cognitive behavio?r* therap*" or "cognitive behavio?r* psychotherap*" or CBT or "cognitive therap*" or "cognitive psychotherap*").tw,kw,kf. |
| 18 | (acceptance adj3 therap*).tw,kw,kf. |
| 19 | ("behavio?ral intervention*" or "compassion-focused therap*" or "compassion focused therap*" or "cognitive analytic therap*" or "couples therap*" or "behavio?al activation" or "social cognition").tw,kw,kf. |
| 20 | "positive behavio?r* intervention* and support*".tw,kw,kf. |
| 21 | or/14-20 |
| 22 | 13 and 21 |
| 23 | "quality of life"/ |
| 24 | ("psychological wellbeing" or "psychological well-being" or "emotional wellbeing" or "emotional well-being").tw,kw,kf. |
| 25 | ("life quality" or "quality of life" or "quality adjusted life year*" or "health status" or "health level" or "level of health" or "mental health" or "quality adjusted survival").tw,kw,kf. |
| 26 | or/23-25 |
| 27 | 22 and 26 |
| 28 | limit 27 to english language |
